# Supplementary material for: Oral 4‘fluorouridine provides postexposure protection against lethal Nipah virus infection
Source: bioRxiv. 2026 Feb 22:2026.02.21.707194. Preprint. [Version 1] doi: 10.64898/2026.02.21.707194 (PMC12934678; doi:10.64898/2026.02.21.707194)
Supplement: Supplement 1 [file media-1.pdf]

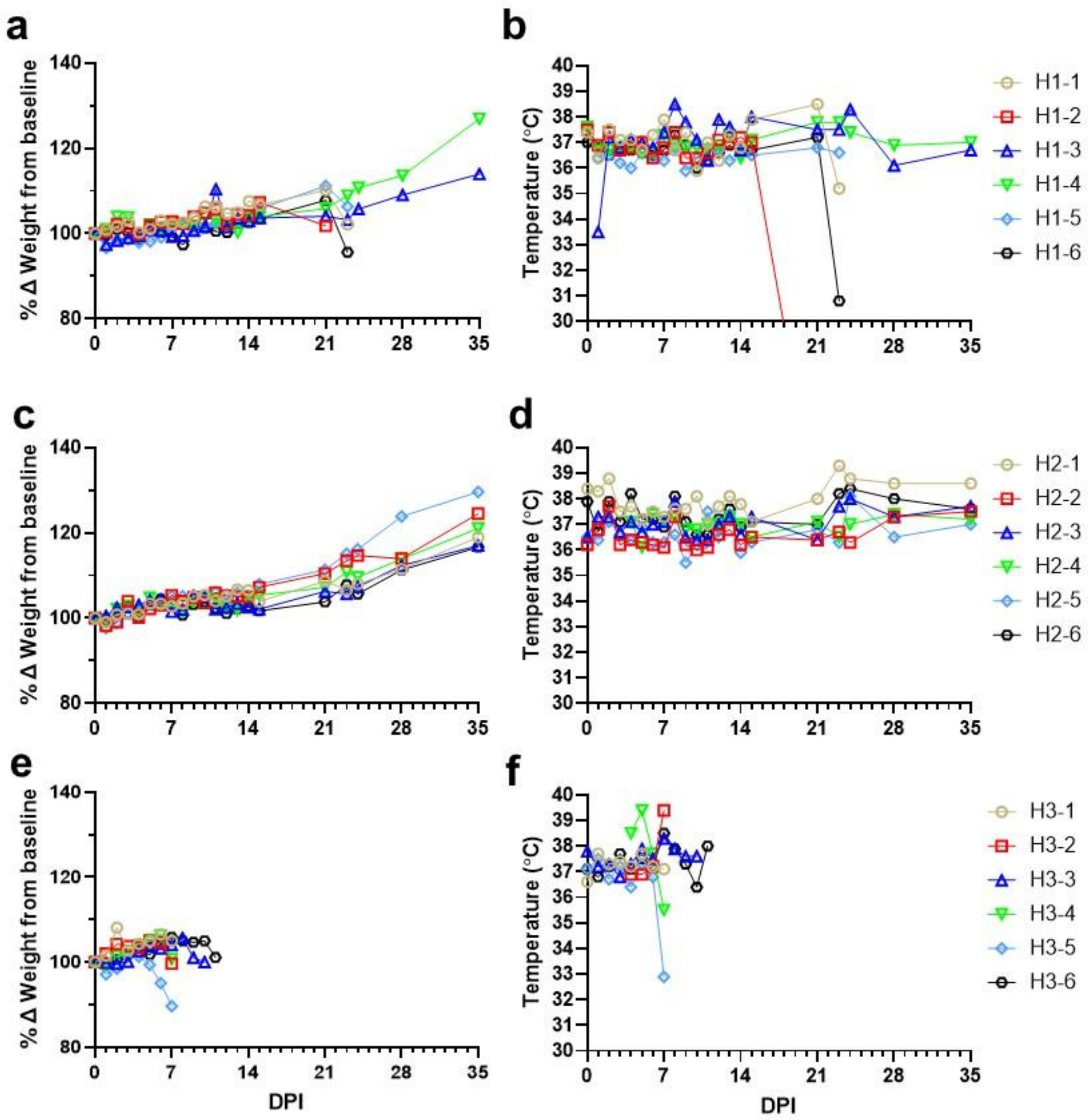

Supplementary Figure 1: Weight and temperature monitoring of hamsters following challenge with NiVB and treatment with 4`-FIU. Weight was measured at the indicated timepoints **(a, c, e)**. Temperature was monitored using telemetry and scanned at the indicated timepoints **(b, d, f)**.
